# High-Efficiency Strategy for Reducing Decomposition Potential of Lithium Formate as Cathode Prelithiation Additive for Lithium-Ion Batteries

**DOI:** 10.3390/nano15161225

**Published:** 2025-08-11

**Authors:** Yaqin Guo, Ti Yin, Zeyu Liu, Qi Wu, Yuheng Wang, Kangyu Zou, Tianxiang Ning, Lei Tan, Lingjun Li

**Affiliations:** School of Materials Science and Engineering, Changsha University of Science and Technology, Changsha 410114, China; 202228020413@stu.csust.edu.cn (Y.G.); 202228020409@stu.csust.edu.cn (T.Y.); 202228020403@csust.edu.cn (Z.L.); qiwu@stu.csust.edu.cn (Q.W.); 202302140242@stu.csust.edu.cn (Y.W.); ningtianxiang@csust.edu.cn (T.N.); tanlei@csust.edu.cn (L.T.); lingjun.li@csust.edu.cn (L.L.)

**Keywords:** lithium-ion batteries, cathode prelithiation, lithium formate, reducing reaction barrier, electrochemical properties

## Abstract

Lithium-ion batteries (LIBs) have attracted extensive attention as a distinguished electrochemical energy storage system due to their high energy density and long cycle life. However, the initial irreversible lithium loss during the first cycle caused by the formation of the solid electrolyte interphase (SEI) leads to the prominent reduction in the energy density of LIBs. Notably, lithium formate (HCOOLi, LFM) is regarded as a promising cathode prelithiation reagent for effective lithium supplementation due to its high theoretical capacity of 515 mAh·g^−1^. Nevertheless, the stable Li-O bond of LFM brings out the high reaction barrier accompanied by the high decomposition potential, which impedes its practical applications. To address this issue, a feasible strategy for reducing the reaction barrier has been proposed, in which the decomposition potential of LFM from 4.84 V to 4.23 V resulted from the synergetic effects of improving the electron/ion transport kinetics and catalysis of transition metal oxides. The addition of LFM to full cells consisting of graphite anodes and LiNi_0.834_Co_0.11_Mn_0.056_O_2_ cathodes significantly enhanced the electrochemical performance, increasing the reversible discharge capacity from 156 to 169 mAh·g^−1^ at 0.1 C (2.65–4.25 V). Remarkably, the capacity retention after 100 cycles improved from 72.8% to 94.7%. Our strategy effectively enables LFM to serve as an efficient prelithiation additive for commercial cathode materials.

## 1. Introduction

With the depletion of non-renewable energy sources and the intensification of environmental pollution, the development of renewable and clean energy has received widespread attention [1,2,3]. However, most renewable energy sources exhibit inherent intermittency, geographical constraints, and uncontrollable variability, posing significant challenges to their widespread adoption. Consequently, researchers have shifted their focus toward developing clean and high-efficient energy storage systems. Among these, lithium-ion batteries (LIBs) have emerged as one of the most widely utilized electrochemical energy storage technologies due to their high energy density, long cycle, and absence of memory effects [4,5]. Nevertheless, during the initial charging process, the formation of a solid electrolyte interphase (SEI) layer on the anode leads to irreversible lithium loss, which directly reduces the overall energy density and operational lifespan of LIBs [6,7,8,9]. 

To address this critical issue, prelithiation strategies have been proposed as a viable solution, owing to their ability to provide additional lithium sources [10,11,12,13]. Generally, prelithiation techniques can be classified into cathode prelithiation [14,15,16,17,18,19,20] and anode prelithiation [21,22,23,24]. Common anode prelithiation reagents include metallic lithium, lithiated alloys, in which metallic lithium and lithiated alloys offers exceptionally high compensation capacities [25]. However, the high reactivity of metallic lithium inevitably brings out the safety risks. Moreover, lithiated alloys release Li^+^ through irreversible conversion reactions between transition metals and lithium-containing compounds, which leads to remaining metal oxides in the battery system, thus reducing the overall energy density. It is noticed that cathode prelithiation has emerged as the most promising strategy due to its convenience, safety, and low cost. Moreover, cathode prelithiation is compatible with the existing manufacturing processes of LIBs and is suited for large-scale applications. 

Among the various cathode prelithiation additives [26,27,28], lithium formate (LFM), as an organic lithium salt, exhibits a high theoretical specific capacity of 515 mAh·g^−1^ [29,30], but the stable Li-O bonds in LFM induce high reaction barriers and elevated decomposition potentials, significantly exceeding the normal operating voltage range of cathodes. However, an excessively high potential may trigger electrolyte decomposition, thereby deteriorating the electrochemical performance. In the present study, the decomposition potential of LFM was effectively reduced through a strategy involving both enhanced electron/ion transport kinetics and decreased reaction activation energy [31,32]. The transport properties were improved through particle size reduction and optimized conductive agent selection, achieving near-complete capacity utilization efficiency (~100%). Furthermore, particular emphasis was placed on investigating the catalytic effects of transition metal oxides (NiO [33,34,35,36], Co_3_O_4_ [37], and Mn_2_O_3_ [38]), which are the key constituent elements of ternary cathode materials, to reduce the reaction activation energy. The synergistic combination of conductive agents and catalysts successfully decreased the decomposition potential from 4.84 to 4.23 V. Remarkably, LFM incorporation substantially improved the first-cycle discharge specific capacity of full cells employing graphite and NCM834 from 156 to 169 mAh·g^−1^. Our strategy effectively enables the practical application of LFM as an appropriate prelithiation additive for commercial cathode materials.

## 2. Materials and Methods

### 2.1. Materials

Polyvinylidene fluoride (PVDF, AR), carboxymethyl cellulose (CMC) NiO (AR, 99%), Co_3_O_4_ (99.9%), and Mn_2_O_3_ (98%) were purchased from Aladdin (Shanghai, China). Lithium formate (LFM, 99.9%) and LiOH (AR, 98%) were purchased from Macklin (Shanghai, China). Acetylene black (AB, AR), Super P (SP, AR), Ketjen black (KB, AR), and graphite (AR) were purchased from Canrd (Guangdong, China).

### 2.2. Preparation of B-LFM with Decreased Particle Size

LFM was prepared using a planetary ball mill (MSK-SFM-15, Shenzhen Kejing, China), with a ball-to-powder ratio of 5:1 and a particle size distribution ratio of zirconia grinding media of 3:3:5 (large/medium/small). An amount of 1.9 g of commercial LFM (named C-LFM) was loaded into the milling jar with the zirconia balls. The ball-milling process was performed by rotating the sample forward at 600 rpm for 30 min followed by rotation at 200 rpm for 15 min. Subsequently, the rotation direction was reversed at 600 rpm for 30 min and then maintained at 200 rpm for another 15 min. This complete milling cycle was repeated three times to obtain the final LFM after ball milling (named B-LFM).

### 2.3. Preparation of LiNi_0.834_Co_0.11_Mn_0.056_O_2_ (NCM834) Cathode Material

The NCM834 was synthesized via a solid-state reaction with a total stoichiometric amount of 0.03 mol LiOH and Ni_0.834_Co_0.11_Mn_0.056_(OH)_2_ and a lithium excess of 5% (LiOH:Ni_0.834_Co_0.11_Mn_0.056_(OH)_2_ = 1.05:1). The mixed powders were homogenized by grinding in an agate mortar for 0.5 h and then subjected to a two-step calcination process in a tube furnace (OTF-1200X-S50, Shenzhen Kejing, China)under oxygen atmosphere, where the temperature was first raised from room temperature to 480 °C at a heating rate of 5 °C·min^−1^ and maintained for 5 h, and then continued to 750 °C at the same rate with a 12 h holding period.

### 2.4. Preparation of LFM/Catalyst Composite Electrodes and Pure Conductive Additive Electrodes

The pure C-LFM and B-LFM electrodes were fabricated with active materials (60 wt.%), conductive agents (30 wt.%), and polyvinylidene fluoride (PVDF, 10 wt.%). Three conductive agents, acetylene black (AB), Super P (SP), and Ketjen black (KB), were employed. The pure conductive carbon electrode was composed of 75 wt.% conductive carbon and 25 wt.% PVDF. The LFM/catalyst composite electrodes were prepared by blending B-LFM, catalysts (NiO, Co_3_O_4_, and Mn_2_O_3_), AB, and PVDF. The mass ratios of B-LFM to the catalysts were 5:1, 4:2, and 3:3, respectively, and the corresponding electrodes were named B-LFM/catalyst-1, B-LFM/catalyst-2, and B-LFM/catalyst-3.

### 2.5. Preparation of NCM/B-LFM/Catalyst Composite Electrodes and Graphite Electrodes

The pristine electrode was fabricated by mixing NCM834 (80 wt.%), AB (10 wt.%), and PVDF (10 wt.%), while the modified electrode (NCM834/NiO/B-LFM) was prepared by blending the NCM834, B-LFM, NiO, AB, and PVDF with mass ratios of 6:1 between the NCM834 and B-LFM and 4:2 between the lithium formate and NiO. The graphite electrode was composed of 70 wt.% graphite, 15 wt.% AB, and 15 wt.% carboxymethyl cellulose (CMC).

### 2.6. Material Characterization

X-ray diffraction (Bruker D8 Endeavor, Bruker, New York, USA, λ = 0.154 nm) was used to study the crystal structure of the materials. The morphology and particle size of the samples were observed by scanning electron microscopy (SEM, JSM-7900F, JEOL, Ltd. Japan). The specific surface area of the samples was determined by the Brunauer–Emmett–Teller (BET, ASAP2460, Micromeritics, GA, USA) method. The particle size (NanoBrook Omni, Bruker, New York, NY, USA) of the sample was visually tested through a particle size tester.

## 3. Results

### 3.1. Impact of Conductive Additive on Delithiation Potential of LFM

**Figure 1:**
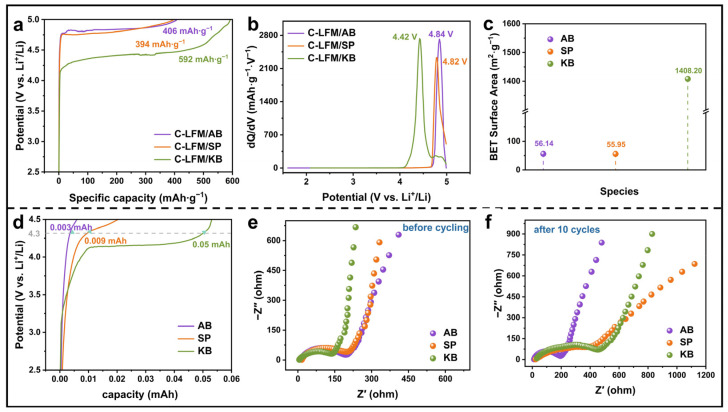
(**a**) The galvanostatic charge–discharge (GCD) profiles of C-LFM/AB, C-LFM/SP, and C-LFM/KB at 0.1 C within 5 V potential. (**b**) dQ/dV curves of C-LFM/AB, C-LFM/SP, and C-LFM/KB. (**c**) Detailed values of specific surface areas of AB, SP, and KB. (**d**) GCD of pure AB, SP, and KB electrodes at 0.1 C within 4.5 V potential. (**e**) Nyquist plots of pure AB, SP, and KB electrodes before cycling. (**f**) Nyquist plots of pure AB, SP, and KB electrodes after 10 cycles.

The conductivity of electrodes critically determines the electrochemical performances of active materials due to the regulated electron transport efficiency, interfacial reaction kinetics, and polarization degree [39,40,41,42]. Notably, LFM has poor conductivity; therefore, highly efficient conductive agents should be added to ensure the decomposition of LFM under an appropriate voltage range. Thus, three conductive agents (AB, SP, and KB) have been explored for the influence of the delithiation potential of C-LFM. As shown in Figure 1a, the decomposition of C-LFM can be realized by the addition of three conductive agents under a 0.1 C rate with a 5 V cutoff voltage (1 C = 515 mAh·g^−1^). Moreover, the decomposition potentials of C-LFM/AB, C-LFM/SP, and C-LFM/KB are 4.84, 4.82, and 4.42 V, respectively (Figure 1b). Notably, although the KB conductive agent can bring out the lower decomposition potential of C-LFM, its specific capacity exceeds the theoretical value of LFM, suggesting the possible side reactions triggered by the large specific surface area of KB. As shown in Figure 1c, the discrepancy of the specific surface areas about the three conductive agents have been verified by BET tests. Among them, KB exhibits the largest specific surface area (1408.2 m^2^ g^−1^), which potentially leads to the side reactions. To further demonstrate the abnormal capacity of C-LFM/KB, the pure KB electrode has been explored. As depicted in Figure 1d, the pure KB electrode delivers the highest capacity (0.05 mAh) due to the pronounced side reactions, which can be attributed to its extensive specific surface area. Meanwhile, the pure AB electrode demonstrates minimal parasitic reactions, and the specific surface area of AB is 56.14 m^2^·g^−1^, which illustrates that AB is a suitable conductive additive. Moreover, electrochemical impedance spectroscopy (EIS) results reveal that KB exhibits relatively low initial impedance but undergoes a substantial increase after 10 cycles, indicating the progressive degradation of its conductive network (Figure 1e,f). Conversely, AB maintains exceptional charge-transfer characteristics throughout cycling, demonstrating remarkable stability in charge-transfer resistance (R_ct_). To sum up, AB was employed as the optimal conductive additive for LFM electrode systems.

### 3.2. Impact of Particle Size on Delithiation Potential of LFM

**Figure 2:**
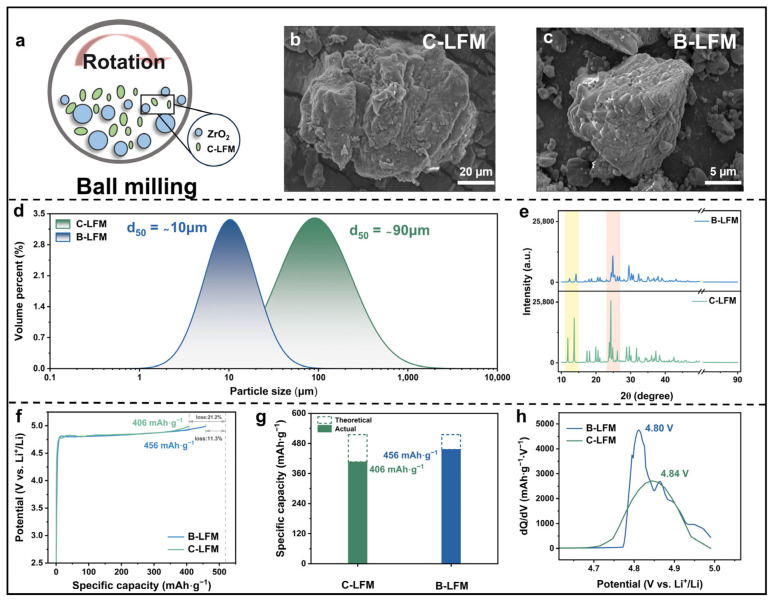
(**a**) Schematic diagram of ball milling of C-LFM. SEM images of (**b**) C-LFM and (**c**) B-LFM. (**d**) Particle size distributions of C-LFM and B-LFM. (**e**) XRD patterns of C-LFM and B-LFM. (**f**) The GCD of C-LFM and B-LFM at 0.1 C within 5 V potential. (**g**) The actual and theoretical specific capacity bar chart of C-LFM and B-LFM. (**h**) The dQ/dV curves of C-LFM and B-LFM.

The reduction in particle size can effectively facilitate internal electron transport and decrease the reaction energy barrier, thereby decreasing the decomposition potential [43,44,45]. As illustrated in Figure 2a, ball milling was employed to achieve particle refinement. Figure 2b,c present the morphological characteristics of C-LFM and B-LFM, as observed by SEM. Furthermore, dynamic light scattering (DLS) analysis (Figure 2d) confirmed the reduced particle size distribution of LFM, with the average diameter decreasing from ~90 µm to ~10 µm. The X-ray diffraction (XRD) patterns in Figure 2e verify the well-preserved crystalline structure of B-LFM, with all characteristic peaks maintained. The observed decrease in the peak intensity and broadening of full width at half maximum (FWHM) for B-LFM provide direct evidence of particle size reduction, in excellent agreement with the Scherrer equation. Importantly, no impurity peaks were detected, indicating that the ball-milling process did not introduce any contaminants. As depicted in Figure 2f,g, electrochemical evaluation through galvanostatic cycling at 0.1 C (1 C = 515 mAh·g^−1^) with a 5 V cutoff voltage reveals that B-LFM achieved a significantly enhanced initial capacity of 456 mAh·g^−1^, representing an 88.7% decomposition efficiency compared to C-LFM (406 mAh·g^−1^). Furthermore, as shown in Figure 2h, B-LFM exhibits a lower delithiation potential (4.80 V), indicating improved reaction kinetics resulting from the increased interfacial contact area between the active material and acetylene black. These comprehensive results indicate that reducing the particle size through ball milling can synergistically enhance the electrochemical performance. Specifically, it can not only lower the decomposition potential by promoting the transport of lithium ions but also significantly increase the initial specific capacity by improving the interfacial kinetics.

### 3.3. Impact of Catalyst on Delithiation Potential of LFM

**Figure 3:**
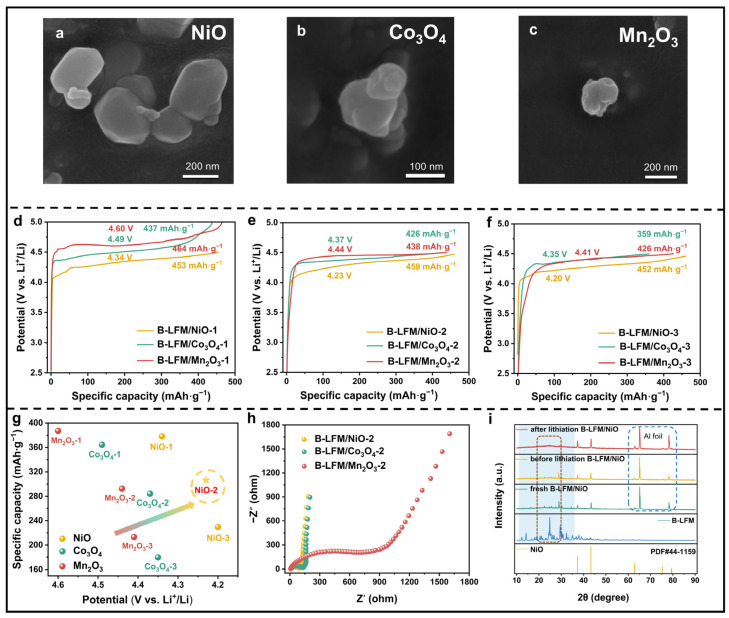
SEM images of (**a**) NiO, (**b**) Co_3_O_4_, and (**c**) Mn_2_O_3_. The GCD of (**d**) B-LFM/catalyst-1, (**e**) B-LFM/catalyst-2, and (**f**) B-LFM/catalyst-3. (**g**) Comparison of decomposition voltages and actual specific capacities of catalysts in different proportions. (**h**) Nyquist plots of B-LFM/catalyst-2 after 10 cycles. (**i**) XRD patterns of B-LFM/NiO-2 during the electrochemical process.

The attenuation potential was reduced to 4.80 V through the B-LFM/AB; however, this value remains prohibitively high for practical compatibility with commercial cathode materials. Given the ability of catalysts to lower reaction activation energy and enhance electrochemical activity [46,47], three catalysts (NiO, Co_3_O_4_, and Mn_2_O_3_) were systematically evaluated at 5:1, 4:2, and 3:3 ratios (catalyst-1, catalyst-2, and catalyst-3) for their effects on B-LFM decomposition. Prior to testing, the commercial catalysts were ball-milled to achieve homogeneous particle sizes within 200–400 nm, with DLS (Figure S3) confirming average diameters of 415 (NiO), 218 (Co_3_O_4_), and 245 nm (Mn_2_O_3_). The SEM images in Figure 3a–c confirm the morphological integrity of the ball-milled catalysts, and the XRD patterns in Figure S4 demonstrate that ball milling preserved their crystalline structure, as evidenced by the diffraction patterns matching standard reference spectra. Among these catalysts, NiO exhibited an excellent catalytic performance at all ratios (Figure 3d–f), and only B-LFM/NiO-2 and B-LFM/NiO-3 achieved B-LFM decomposition at voltages below 4.3 V. Although increasing the NiO from B-LFM/NiO-2 to B-LFM/NiO-3 further reduces the voltage by 0.03 V, this marginal improvement (Figure 3g) comes at the cost of a significant 76 mAh·g^−1^ capacity loss, establishing B-LFM/NiO-2 as the optimal formulation. Remarkably, NiO delivers the lowest decomposition potential of 4.23 V (Figure S5) despite its relatively larger particle size, highlighting its exceptional catalytic activity. As illustrated in Figure 3h, B-LFM/NiO-2 exhibits the lowest charge-transfer impedance after 10 cycles. The complete disappearance of B-LFM diffraction peaks in the XRD analysis (Figure 3i) provides definitive evidence for thorough B-LFM decomposition and lithium replenishment. These comprehensive results unequivocally identify NiO-2 as the most effective catalyst for simultaneously lowering the operational voltage and enhancing the electrochemical performance in B-LFM systems.

### 3.4. Electrochemical Properties of Half Cell

**Figure 4:**
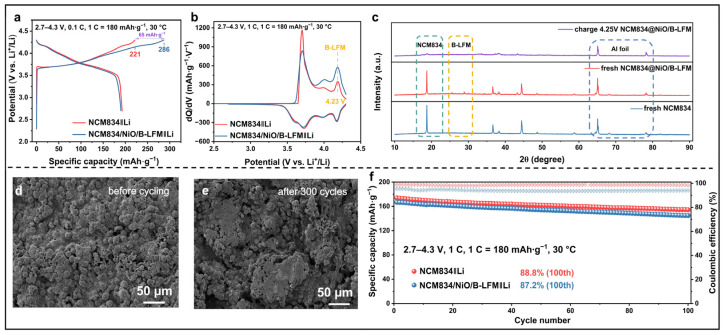
(**a**) The GCD of NCM834‖Li and NCM834/NiO/B-LFM‖Li at 0.1 C (potential window: 2.7–4.3 V). (**b**) The dQ/dV curves of NCM834‖Li and NCM834/NiO/B-LFM‖Li. (**c**) XRD patterns of modified electrode before and after cycling. SEM images of modified NCM834/NiO/B-LFM electrode (**d**) before and (**e**) after cycling. (**f**) Cycling performances of NCM834‖Li and NCM834/NiO/B-LFM‖Li at 1 C.

To evaluate the practicability of B-LFM as a lithium compensation additive, the electrochemical performance was systematically investigated using an NCM834 cathode material in a half cell. The first three cycles were conducted at a current density of 0.1 C (1 C = 180 mAh·g^−1^), followed by cycling at 1 C, with a voltage window of 2.7–4.3 V. As shown in Figure 4a, the NCM834/NiO/B-LFM‖Li demonstrated significant improvement compared to the NCM834‖Li. The modified system (NCM834/NiO/B-LFM‖Li) exhibited LFM decomposition initiating at approximately 4 V, accompanied by a substantial increase in the first-cycle charge capacity from 221 to 286 mAh·g^−1^, confirming an effective lithium compensation of 65 mAh·g^−1^. The dQ/dV analysis further verified the onset potential of B-LFM decomposition at ~4 V (Figure 4b). The complete disappearance of characteristic diffraction peaks in Figure 4c confirm the full consumption of B-LFM and its role in active lithium replenishment. As revealed in Figure 4d,e and Figure S6, B-LFM decomposition led to increased electrode surface roughness and structural inhomogeneity, partially compromising the electrode integrity. In Figure 4f, these morphological changes correlate with the observed minor capacity fading in the cycling performances of the modified samples. Collectively, these results provide compelling evidence that B-LFM serves as a prelithiation agent to mitigate the capacity loss in the NCM834 cathode, although the morphological alterations induced by its decomposition require further optimization to enhance the cycling stability.

### 3.5. Electrochemical Properties of Full Cell

**Figure 5:**
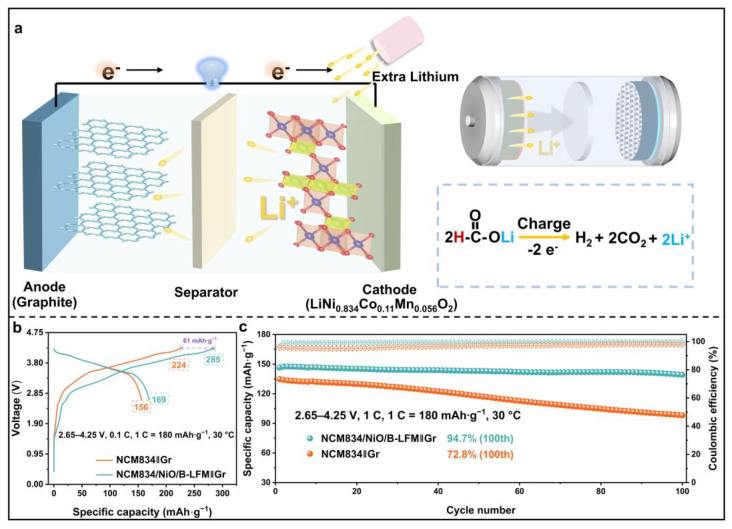
(**a**) Lithium replenishment mechanism and schematic diagram of LIBs. (**b**) The GCD of NCM834‖Gr and NCM834/NiO/B-LFM‖Gr at 0.1 C (cell voltage window: 2.65–4.25 V). (**c**) Cycling performances of NCM834‖Gr and NCM834/NiO/B-LFM‖Gr at 1 C.

Furthermore, to validate the feasibility of B-LFM as a lithium compensation agent, full cells were assembled using graphite and NCM834 for electrochemical evaluation. A schematic illustration of the lithium compensation mechanism was employed in this study and is shown in Figure 5a. Electrochemical testing (Figure 5b) revealed that the NCM834‖Gr full cell exhibited an initial charge specific capacity of 224 mAh·g^−1^, which increased to 285 mAh·g^−1^ upon the incorporation of B-LFM. The full cells were initially cycled at 0.1 C for three formation cycles, followed by 100 cycles at 1 C. As shown in Figure 5c, the control NCM834‖Gr full cell retained only 97 mAh·g^−1^ after 100 cycles with a capacity retention of 72.6%, while NCM834/NiO/B-LFM‖Gr maintained 138 mAh·g^−1^ with a significantly improved capacity retention of 94.7%. Meanwhile, the TEM characterization presents compelling evidence that the incorporation of B-LFM effectively suppresses the structural degradation of NCM834 after cycling, in which the modified electrodes display substantially fewer and thinner degradation layers compared to their unmodified electrodes (Figure S7). This marked improvement in structural stability highlights the crucial role of B-LFM in maintaining the electrode integrity during electrochemical cycling. These results demonstrate that B-LFM not only enhances the specific capacity but also preserves the cycling stability of full cells. The electrochemical findings collectively confirm the viability of B-LFM as a prelithiation additive for full cell cathodes, effectively improving the overall cell capacity.

## 4. Discussion and Conclusions

In summary, the selection of conductive additives was found to significantly influence the lithiation potential. Considering key factors such as the attenuation potential, specific surface area, and impedance variations, AB was adopted as the optimal conductive additive. Furthermore, since the particle size of LFM directly affects the reaction kinetics, we employed ball milling to reduce its particle size, resulting in an increased decomposition efficiency from 78.8% to 88.7%. Additionally, the type and ratio of the catalyst played a crucial role in modulating the electrochemical performance. Notably, the introduction of B-LFM/NiO-2 effectively lowered the lithiation potential from 4.84 V to 4.23 V. When LFM was utilized as a lithium-supplementing cathode additive, the tests of the half cell confirmed its excellent compatibility with the NCM834 cathode. Despite observable surface damage to the electrode, the integrity and functionality of the entire battery system were maintained. More importantly, full cell evaluations demonstrated substantial improvements in both the capacity and cycling stability. These findings successfully demonstrate the enhanced prelithiation capability of LFM as a high-performance cathode additive, offering a promising strategy for advanced lithium-ion battery systems.

## Data Availability

The original contributions presented in this study are included in the article/Supplementary Material. Further inquiries can be directed to the corresponding author.

## Supplementary Materials

The following supporting information can be downloaded at: https://www.mdpi.com/article/10.3390/nano15161225/s1, Figure S1: The SEM images of AB (a), SP (b), KB (c) before cycling. The SEM images of AB (d), SP (e), KB (f) after cycling; Figure S2: The dQ/dV curves of C-LFM and B-LFM; Figure S3: Particle size distributions of NiO, Co_3_O_4_, and Mn_2_O_3_; Figure S4 XRD patterns of NiO, Co_3_O_4_, and Mn_2_O_3_; Figure S5 The dQ/dV curves of B-LFM/catalyst-2; Figure S6: The SEM images of unmodified electrodes in NCM834||Li half cell before (a) and after cycling (b); Figure S7: The TEM images of cycled NCM834 electrodes in NCM834‖Gr (a) and NCM834/NiO/B-LFM‖Gr (b) systems; Table S1: The comprehensive comparison of cathode prelithiation agents.
