# Supplementary material for: High-Efficiency Strategy for Reducing Decomposition Potential of Lithium Formate as Cathode Prelithiation Additive for Lithium-Ion Batteries
Source: Nanomaterials (Basel). 2025 Aug 11;15(16):1225. doi: 10.3390/nano15161225 (PMC12388210; doi:10.3390/nano15161225)
Supplement: Supplementary file 1 [file nanomaterials-15-01225-s001.zip › nanomaterials-3796278-supplementary.pdf]

Supplementary Materials

High-Efficiency Strategy for Reducing Decomposition Potential of Lithium Formate as Cathode Prelithiation Additive for Lithium-Ion Batteries

Yaqin Guo, Ti Yin, Zeyu Liu, Qi Wu, Yuheng Wang, Kangyu Zou *, Tianxiang Ning, Lei Tan and Lingjun Li

School of Materials Science and Engineering, Changsha University of Science and Technology, Changsha 410114, China; 202228020413@stu.csust.edu.cn (Y.G.); 202228020409@stu.csust.edu.cn (T.Y.); 202228020403@csust.edu.cn (Z.L.); qiwu@stu.csust.edu.cn (Q.W.); 202302140242@stu.csust.edu.cn (Y.W.); ningtianxiang@csust.edu.cn (T.N.); tanlei@csust.edu.cn (L.T.); lingjun.li@csust.edu.cn (L.L.)

* Correspondence: ky-zou@csust.edu.cn; +86-181-75148731

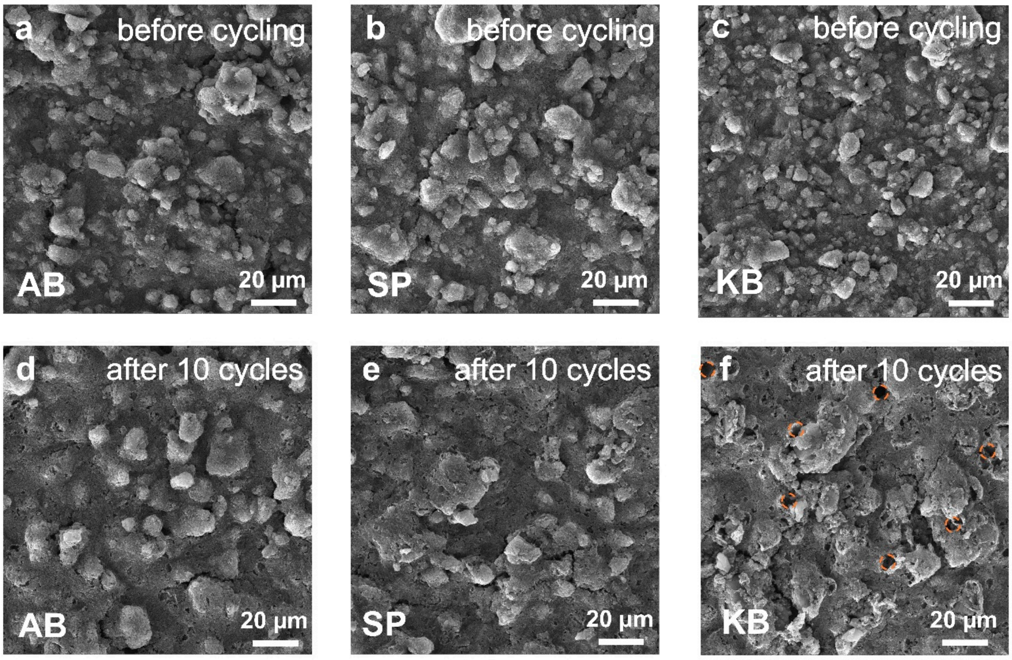

Figure S1. The SEM images of AB (**a**), SP (**b**), KB (**c**) before cycling. The SEM images of AB (**d**), SP (**e**), KB (**f**) after cycling.

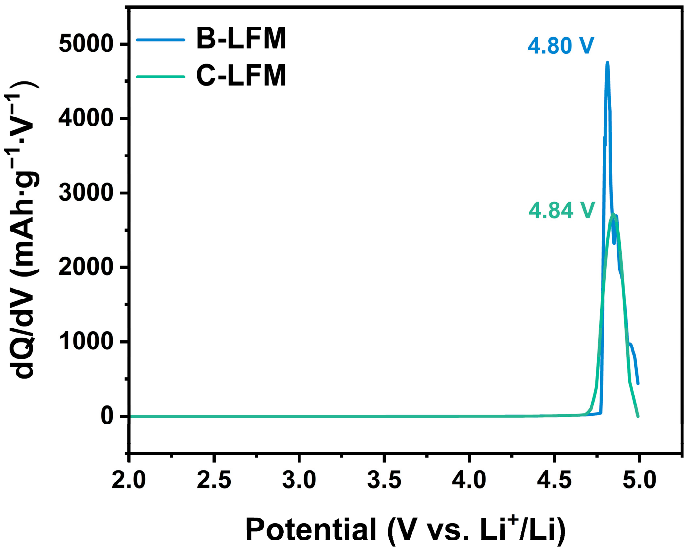

Figure S2. The dQ/dV curves of C-LFM and B-LFM.

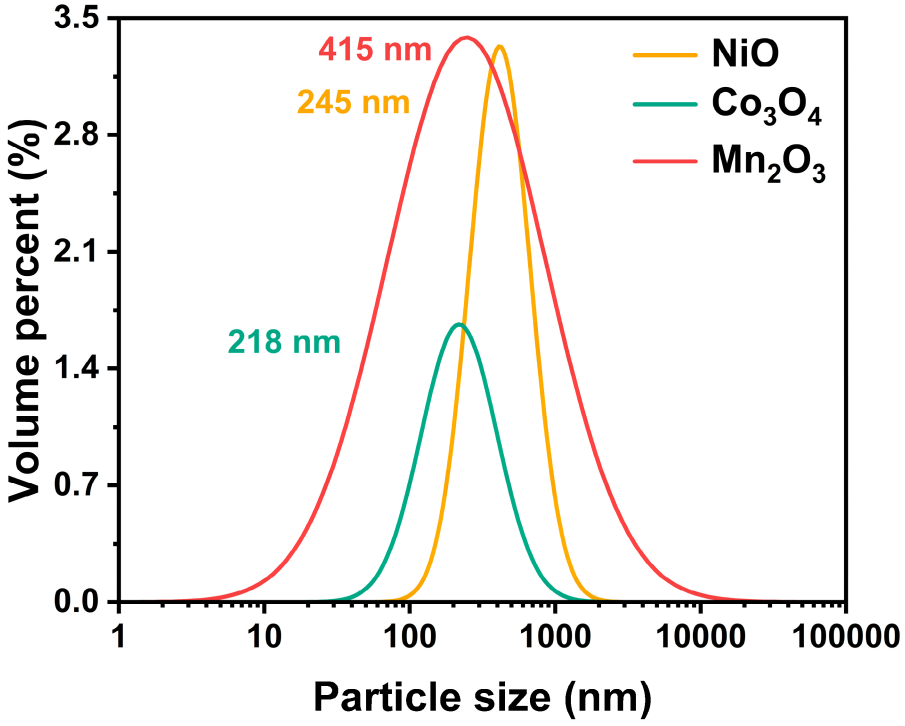

Figure S3. Particle size distributions of NiO, Co_3O_4, and Mn_2O_3.

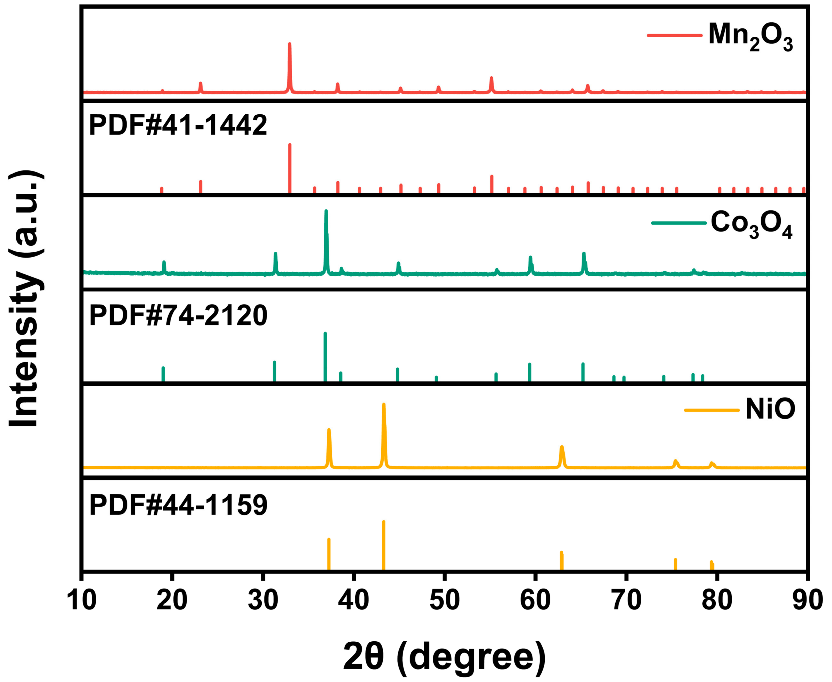

Figure S4. XRD patterns of NiO, Co_3O_4, and Mn_2O_3.

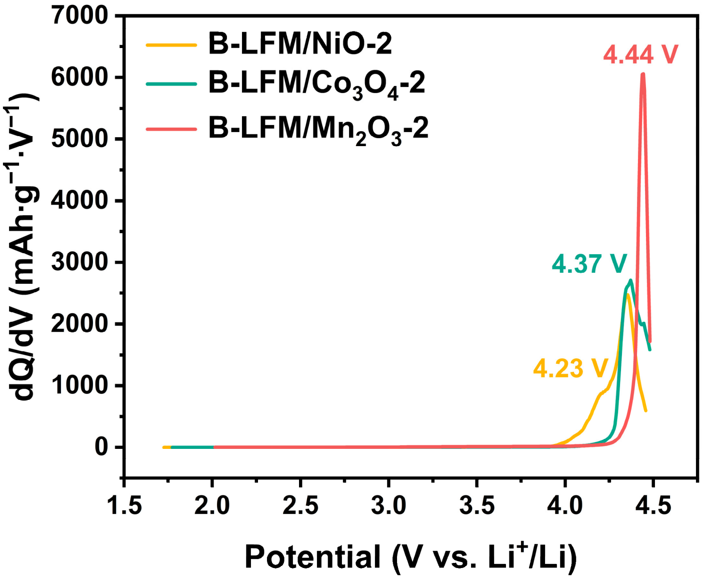

Figure S5. The dQ/dV curves of B-LFM/catalyst-2.

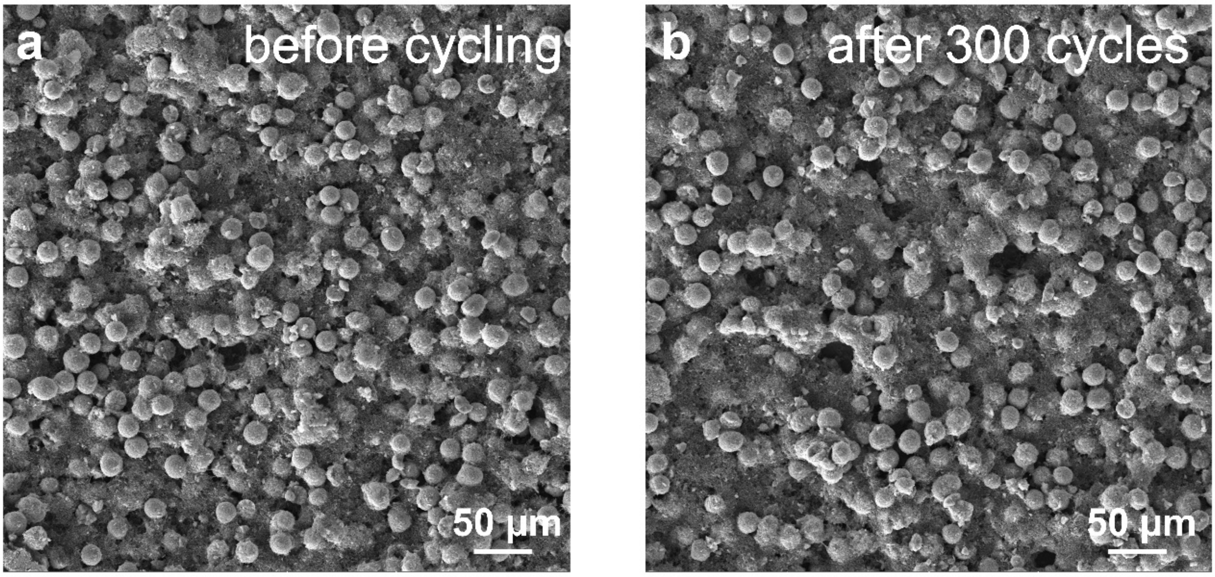

Figure S6. The SEM images of unmodified electrodes in NCM834||Li half cell before (a) and after cycling (b).

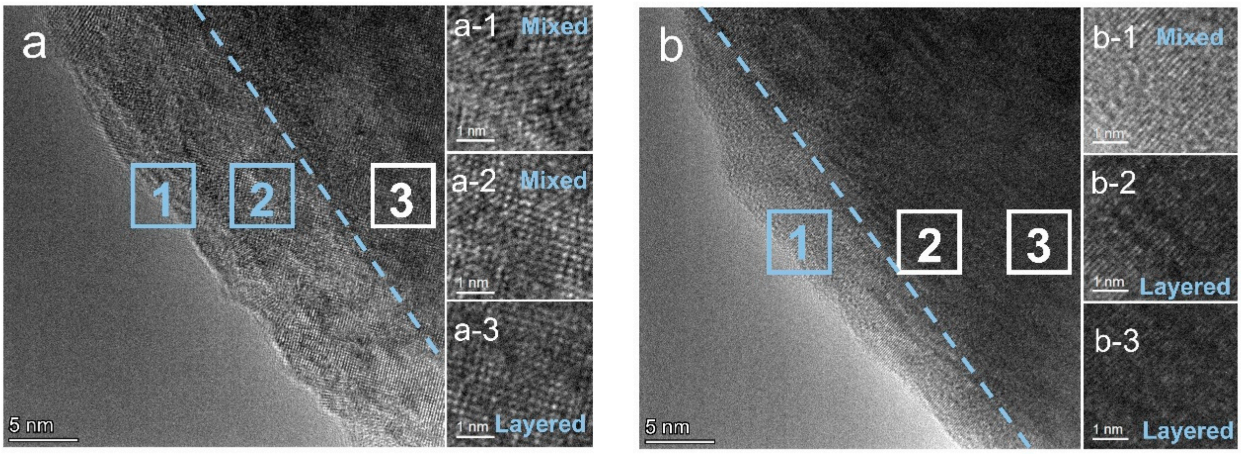

Figure S7. The TEM images of cycled NCM834 electrodes in NCM834‖Gr (a) and NCM834/NiO/B-LFM‖Gr (b) systems.

Table S1. The comprehensive comparison of cathode prelithiation agents.

Prelithiation agent	Potential-reduction strategy	Reduced potential	Lithium release in full cell	Batteries	Capacity retention after 100 cycles	Reference
$Li_2C_4O_4$	Reduced particle size and prelithiation agent content	4.25 V	30.7 mAh g^{-1}	NMC622+$Li_2C_4O_4$|| Si/Graphite	71.4%	[S1]
Li_2O_2	Reduced particle size	4.37 V	80 mAh g^{-1}	NMC622/LMFP/Li_2O_2||SLP	-	[S2]
Li_5FeO_4	Adding carbon layer	3.8 V	25.87 mAh g^{-1}	Li_5FeO_4/C|| Graphite	73.3% (50 cycles)	[S3]
LiCPON	Composite prelithiate	4.25 V	51.4 mAh g^{-1}	preli-LRM||SiO_x/C	65.1% (50 cycles)	[S4]
LiF/Fe	Reduced particle size and bilayer structured electrode	3.5 V	27.4 mAh g^{-1}	$LiNi_{0.8}Co_{0.1}Mn_{0.1}O_2$/ LiF/Fe || Graphite	87.9% (200 cycles)	[S5]

LFM	Reduced particle size, conductive agent and catalysts	4.23 V	61 mAh g^{-1}	NCM834/NiO/B-LFM‖Graphite	94.7%	This work

Reference

1. Gomez-Martin, A.; Gnutzmann, M.M.; Adhitama, E.; Frankenstein, L.; Heidrich, B.; Winter, M.; Placke, T. Opportunities and Challenges of $Li_2C_4O_4$ as Pre-Lithiation Additive for the Positive Electrode in NMC622||Silicon/Graphite Lithium Ion Cells. *Adv. Sci.* **2022**, *9 (24)*, 2201742. https://doi.org/10.1002/advs.202201742.
2. Zhang, L.; Jeong, S.; Reinsma, N.; Sun, K.; Maxwell, D.S.; Gionet, P.; Yu, T. Decomposition of Li_2O_2 as the Cathode Prelithiation Additive for Lithium-Ion Batteries without an Additional Catalyst and the Initial Performance Investigation. *J. Electrochem. Soc.* **2021**, *168 (12)*, 120520. https://doi.org/10.1149/1945-7111/ac3e46.
3. Liu, X.; Liu, J.; Peng, J.; Cao, S.; Hu, H.; Chen, J.; Lei, Y.; Tang, Y.; Wang, X. Addressing the Initial Lithium Loss of Lithium Ion Batteries by Introducing Pre-Lithiation Reagent Li_5FeO_4/C in the Cathode Side. *Electrochimica Acta* **2024**, *481*, 143918. https://doi.org/10.1016/j.electacta.2024.143918.
4. Lu, J.; Wang, Y.; Qiao, Y.; Yang, S.; Cheng, X.; Yang, M.; Zhang, J.; Fu, Z. A High-Efficient Stable Surface-Prelithiated $Li_{1.2}Ni_{0.13}Co_{0.13}Mn_{0.54}O_2$ Cathode Enabled by Sacrificial Lithium Nitrides for High-Energy-Density Lithium-Ion Batteries. *Energy Storage Mater.* **2024**, *66*, 103204. https://doi.org/10.1016/j.ensm.2024.103204.
5. Liu, Y.; Lv, Y.; Li, N.; Jia, T.; Huang, S.; Cai, K.; Ouyang, Y.; Kang, F.; Cao, Y. LiF/Fe Composite for Ni-Rich Cathode Prelithiation: Synthesis, Bilayer Structured Electrode and Lithium Loss Compensation. *Chem. Eng. J.* **2024**, *484*, 149550. https://doi.org/10.1016/j.cej.2024.149550.
